# High failure rate of ChAdOx1-nCoV19 immunization against asymptomatic infection in healthcare workers during a Delta variant surge

**DOI:** 10.1038/s41467-022-29404-3

**Published:** 2022-04-01

**Authors:** Rajat Ujjainiya, Akansha Tyagi, Viren Sardana, Salwa Naushin, Nitin Bhatheja, Kartik Kumar, Joydeb Barman, Satyartha Prakash, Rintu Kutum, Akash Kumar Bhaskar, Prateek Singh, Kumardeep Chaudhary, Menka Loomba, Yukti Khanna, Chestha Walecha, Rizwan Ahmed, Ashutosh Yadav, Archana Bajaj, Gaurav Malik, Sahar Qureshi, Swati Waghdhare, Samreen Siddiqui, Kamal Krishan Trehan, Manju Mani, Rajiv Dang, Poonam Das, Pankaj Dougall, Monica Mahajan, Sudipta Sonar, Kamini Jakhar, Reema Kumar, Mahima Tiwari, Shailendra Mani, Sankar Bhattacharyya, Sandeep Budhiraja, Anurag Agrawal, Debasis Dash, Sujeet Jha, Shantanu Sengupta

**Affiliations:** 1 CSIR-Institute of Genomics and Integrative Biology, New Delhi, 110007 India; 2 Academy of Scientific and Innovative Research (AcSIR), Ghaziabad, 201002 India; 3 Department of Clinical Research, Max Super Speciality Hospital, Saket, New Delhi (A Unit of Devki Devi Foundation), New Delhi, 110017 India; 4 Centre of Excellence in Healthcare, IIIT-Delhi, Delhi, 110020 India; 5 Institute of Endocrinology, Diabetes, and Metabolism, Max Super Speciality Hospital, Saket, New Delhi (A Unit of Devki Devi Foundation), New Delhi, 110017 India; 6 Max Hospital Shalimar Bagh, New Delhi, 110088 India; 7 Max Smart Super Speciality Hospital, New Delhi, 110017 India; 8 Max Hospital, Gurgaon, Haryana 122001 India; 9 Institute of Laboratory Medicine & Transfusion Services, Max Super Speciality Hospital, Saket, New Delhi (A Unit of Devki Devi Foundation), New Delhi, 110017 India; 10 Max Super Speciality Hospital, Saket, New Delhi (A Unit of Devki Devi Foundation), New Delhi, 110017 India; 11 Max Hospital Panchsheel, New Delhi, 110017 India; 12 Translational Health Science and Technology Institute, Faridabad, Haryana 121001 India; 13 Department of Internal Medicine, Max Super Speciality Hospital, Saket, New Delhi, 110017 India

**Keywords:** Viral infection, Epidemiology, SARS-CoV-2

## Abstract

Immunization is expected to confer protection against infection and severe disease for vaccines while reducing risks to unimmunized populations by inhibiting transmission. Here, based on serial serological studies of an observational cohort of healthcare workers, we show that during a Severe Acute Respiratory Syndrome -Coronavirus 2 Delta-variant outbreak in Delhi, 25.3% (95% Confidence Interval 16.9-35.2) of previously uninfected, ChAdOx1-nCoV19 double vaccinated, healthcare workers were infected within less than two months, based on serology. Induction of anti-spike response was similar between groups with breakthrough infection (541 U/ml, Inter Quartile Range 374) and without (342 U/ml, Inter Quartile Range 497), as was the induction of neutralization activity to wildtype. This was not vaccine failure since vaccine effectiveness estimate based on infection rates in an unvaccinated cohort were about 70% and most infections were asymptomatic. We find that while ChAdOx1-nCoV19 vaccination remains effective in preventing severe infections, it is unlikely to be completely able to block transmission and provide herd immunity.

## Abstract (web summary)

The authors study a cohort of healthcare workers in India who received two doses of the ChAdOx-1-nCoV19 vaccine during the Delta wave. Using serological data, they infer that 25% of the cohort were infected within 60 days of vaccination, although there were no severe infections leading to hospitalisations.

## Introduction

Immunization is expected to confer protection against infection and severe disease for vaccinees, while also indirectly protecting unvaccinated populations by reducing transmission. Initial data for SARS-CoV-2 vaccines suggested that break-through infections would be infrequent and be associated with lower viral loads, shorter duration, and low likelihood of transmission^1^. The easing of social restrictions such as universal mask-wearing was critically dependent on the validity of these initial observations. However, the global surge in the transmission of the Delta variant of SARS-CoV-2, including in high vaccination populations such as Israel^2,3^, has led to a reconsideration of the policies^4^. More recent data suggests that Delta infections have higher viral loads, with no difference between vaccinated and unvaccinated^5–8^. We observed that during the period of study there was a huge surge in community transmission. As published recently by our group, with almost half the population infected during the surge, we observed uniformly high exposures across social strata, with very similar seropositivity at the end of the surge, ranging from 86 to 91% across unvaccinated sub-groups that had earlier been very different in their exposure levels^8^. We also found only partial clustering of sequenced breakthroughs in a similar setting, suggesting that the transmission was most likely from the general community^7^. Together, the data strongly argues that frequent breakthrough infections and minimally impeded transmission are a possibility with new variants such as Delta and no sub-group of the population was specifically protected.

In the healthcare settings, transmission from vaccinated healthcare workers (HCWs) to patients is a particular concern since many patients may be at high risk. Vaccine effectiveness studies have typically used symptomatic rtPCR positivity as the primary instrument for determining breakthrough rates. The true rate of infection is likely to be much higher since vaccination is known to reduce symptoms and increase the test-seeking threshold. Serologic indicators of infection can overcome these limitations, with the caveat that we do not fully know about the transmissibility of asymptomatic infections discovered by serologic testing. However, epidemiologic models have clearly shown that such infections contribute substantially to aggregate community transmission^9^.

**Fig. 1:**
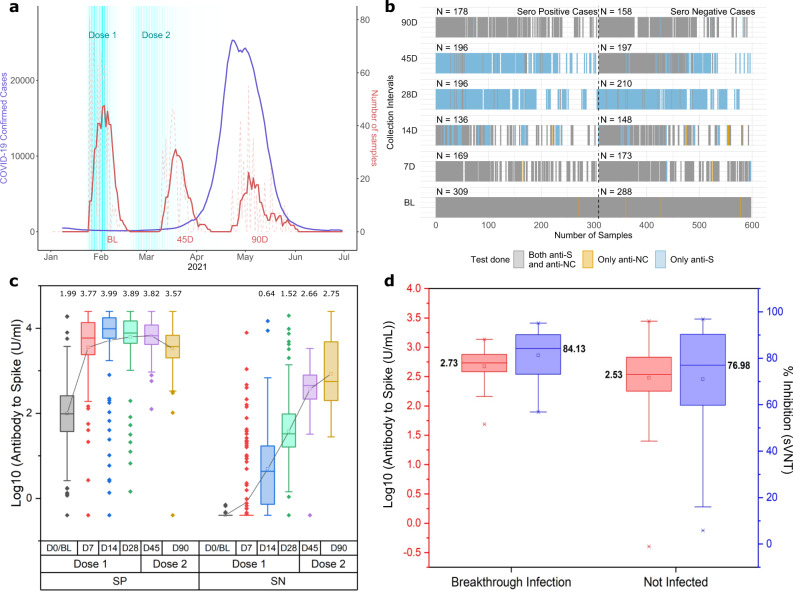
Time period of sample collection and structure of HCW Cohort with Antibody response and kinetics. **a** Time distribution of sampling for HCW cohort shown alongside the Delta-variant driven surge (April, May 2021). Sample collection is 7 days moving average for HCW cohort. **b** Data Structure of the cohort and sampling. **c** Antibody to spike protein in log_10_ units (U/ml) at baseline (D0/BL) and 7, 14, 28,45, 90 days after first dose in baseline antibody naïve (SN) and infection recovered (SP) subjects. Second dose precedes *D*_45_ and *D*_90_ (Number of Samples; For SP at *D*_0_ = 308, *D*_7_ = 162, *D*_14_ = 130, *D*_28_ = 175, *D*_45_ = 103, *D*_90_ = 149 and For SN at *D*_0_ = 281, *D*_7_ = 163, *D*_14_ = 133, *D*_28_ = 196, *D*_45_ = 123, *D*_90_ = 112), Box plot is 25–75 range box, with median line and the square is mean. Whiskers are set at outliers with a coefficient of 1.5. **d** Antibody to Spike in log_10_ units (U/ml) and % Inhibition (sVNT) response for breakthrough cases and non-infected subjects after two doses of vaccine in *D*_0_(Baseline/BL) antibody naïve individuals (*n* = 24 for breakthrough infection group for both anti-S and sVNT and *n* = 71 for not infected group for both anti-S and sVNT). Box plot is 25-75 range box, with median line and the square is mean. Whiskers are set at outliers with a coefficient of 1.5 and cross shapes below and above whiskers are 1 and 99 percentile respectively.

Recently, there was a severe Delta-variant outbreak in Delhi, India^8^. We have shown that the odds of vaccination breakthroughs were greatly increased by Delta-variant, formed larger clusters than seen previously, and were associated with high viral loads^7^. CSIR through its constituent network of research labs and centers spread across the country had initiated cohort-based monitoring of seroprevalence amongst its employees in June 2020^10^. Through follow-up of the same cohort from Delhi-based offices/laboratories, we also found that there was a very high community transmission, with seropositivity in a cohort rising from 42% before the outbreak in Jan–Feb 2020 to 87.4% afterward from June 2021 onwards^8^. Here, through monitoring of two cohorts from Delhi, we provide bridging data for serological estimates of vaccination breakthroughs in double vaccinated HCW cohort, where the Delta outbreak was coincidentally bracketed between serial sample collection time points (Fig. 1a). The structure of the HCW cohort and data available for each time point is shown in Fig. 1b.

## Results and discussion

Briefly, the HCW cohort contained 597 ChAdOx1-nCoV19 vaccine recipients. The timing between the first and second dose varied, but 485 received the second dose within 42 days of the first dose and most subjects received the second dose at 28 ± 3 days. Fifty-two percent of subjects (*n* = 309) had been previously infected with SARS-CoV-2, based on the presence of antibodies to SARS-CoV-2 proteins (spike, anti-S or nucleocapsid, anti-NC) at D_0_, the day of the first dose. There was a robust immunogenic response to two doses of vaccine, irrespective of prior infection (Fig. 1c). Time for seropositivity for antibody naïve subjects from 1st dose of vaccine was 14 days for maximum subjects (~73%) in respect of quantitative antibody response. At day 7, there were 26 subjects (16%), who had a positive quantitative response. On day 28, 97% of subjects had a seroconversion, while 6 subjects did not have seroconversion even on day 28 after the first dose of vaccination.

To have a surrogate matching cohort for calculating infection rates and vaccine effectiveness, the next best matching cohort available to us was the CSIR -Cohort, for which had earlier done two rounds of serosurvey and reported previously^8^. This cohort is not exactly a general population cohort but comprised of researchers, students, scientists, and their family members, many of whom were working as frontline workers in COVID-19 pandemic mitigation and testing work. The seropositivity in Delhi during that period for unvaccinated subjects in our cohort was 87.3 % and that itself we took as an unvaccinated cohort.

Briefly, Phase 3 of CSIR cohort for Delhi was majorly conducted in May-June 2021 matching the period of HCW cohort collection at D90. There were 729 participants during this period of which 637 were seropositive (87.4%). Phase 2 was conducted in January and February of 2021. Of 729 participants in Phase 3, 134 participants had also provided samples in previous phase 2 and were seronegative during that collection time point. Of these 117 were found to be positive in phase 3 (87.3%) and thus were infected during this period.

A single dose in previously infected HCW subjects led to a significant increase in quantitative antibody levels at D7 itself in contrast to baseline antibody naïve subjects where the response was observed at D14. Neutralization levels for antibody naïve subjects had increased after the second dose of vaccination at D28 (Supplementary Fig. 1). However, the neutralization activity (sVNT) was >97% in previously seropositive subjects at day 28 before the second dose and appeared to be saturated after the second dose at D45 and D90. (Fig. 1c, and Supplementary Fig. 1 and Supplementary Note 1).

Since ChAdOx1 immunization only induces Anti-S, subsequent anti-NC seroconversion in previously anti-NC negative subjects was taken as a sensitive and specific marker of new infection^11–13^. Our primary focus was thus on quantifying breakthrough infections in vaccinated subjects, defined as [Anti-NC^negative^ at D_45_ And Anti-NC^positive^ on D_90_] as well as a more relaxed criterion based on doubling of anti-NC and five-fold rise in Anti-S (Supplement Note 2 and Supplementary Fig 2a, b). A sharp increase in Anti-NC concentration after a decline can be taken as a specific marker of reinfection, but the pattern requires a minimum of three consecutive samples and sufficient time for the decline^8^. For baseline seropositive subjects, breakthrough reinfections were assessed based on the aforementioned approach.

Amongst fully vaccinated and uninfected HCWs, i.e. completed 2 weeks beyond the second dose and Anti-NC^negative^ at *D*_45_, the breakthrough infection prevalence at *D*_90_ was 25.3% (95% CI 16.9–35.2%) with 24 of 95 subjects getting an infection. Adjusted protection effectiveness of 70% (95% CI 52–83%)- (Supplementary Method 1 and Supplementary Table 1) for fully vaccinated subjects was observed when adjusted for age and gender. Anti-S concentration or surrogate assays for nAb were poor predictors of vaccination breakthrough (Fig. 1d). We also utilized relaxed criteria, at *D*_90,_ where the CoI at *D*_90_ could be between 0.2 and 1, instead of CoI > =1, and, should show an Anti-NC increase greater than two-fold and Anti-S increase greater than five-fold to qualify as a breakthrough. Using this relaxed criterion to determine infection, adjusted vaccine effectiveness fell to 60% (95% CI 42–76%). These estimates are similar to reports from elsewhere, ranging from 67 to 79%^14–16^.

We observed an adjusted vaccine effectiveness of 45% (95% CI 16–73%) for a single dose, using a similar methodology. Model-based evaluation to study the effect of confounders such as age and gender for fully and partially vaccinated subjects did not lead to substantial changes in the estimates (Supplementary Method 1)

Interestingly, HCWs who were previously infected and had two doses of vaccine had a reinfection rate of only 2.5% over the same period. This corresponds to protection effectiveness of greater than 99%. Higher effectiveness of hybrid immunity induced by vaccination in convalescent subjects has also been reported by the Zoe Covid Study group from the UK^17,18^. The questionnaire survey indicated that there were no severe infections leading to hospitalizations in either of the two cohorts.

**Fig. 2:**
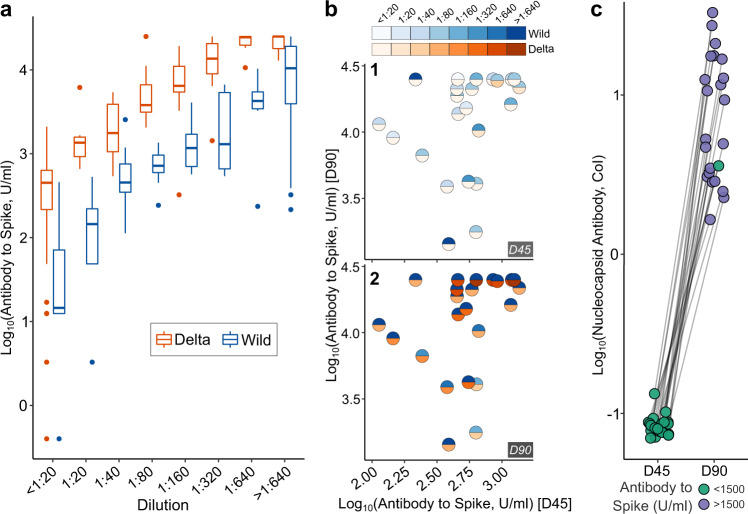
Neutralization to Delta and Wild-type variant w.r.t antibody levels. **a** Box plot for Anti-S titers for similar neutralization titers against Wild type and Delta strain (*n* = 111). Box plot is 25–75 range box, with median line. Whiskers are set at 1.5 * IQR. **b** MnT assay titer to the Wild strain and Delta strain response at day 45 (2b1) and 90 (2b2) for breakthrough cases (*n* = 23, for 1 sample; q.n.s.). They show weak neutralization to both wild type and delta at D45 (before being infected) indicated through light shades in 2b1. However, post-infection at day 90, the same subjects show an increased ability of neutralization to delta strain (Fig. 2b2). **c** Anti-NC antibody response at D45 and D90 when comparing the rise in quantitative titers for breakthrough cases for same subjects in Fig. 2b (*n* = 23).

Our observations should be generalizable to other populations facing a Delta surge. ChAdOx1-nCoV19 immunization in our cohort yielded about the same degree of the humoral immune response as has been reported elsewhere in terms of Anti-S levels^19–21^. Further, Anti-S levels correlated well with MnT assay to both WT and Delta. As expected, neutralization activity for equivalent levels of Anti-S was much lower for Delta than WT, and conversely, higher Anti-S levels were required for comparable neutralization of Delta (Fig. 2a). This is consistent with our previous in vitro observations^7^. Neutralization titers of 1:80 or better against the Delta variant required anti-S levels of over 1500 U/ml. In our data, only 2.4% (3 of 125) subjects who were Anti-NC^negative^ at *D*_0_ and *D*_45_ had Anti-S levels higher than 1500 U/ml at *D*_45_, compared to 96.4% (162 of 168) of subjects who were Anti-NC^positive^ at *D*_0_. Figure 2b shows neutralization activity to Delta and non-Delta before (2b1 at D45) and after breakthrough (2b2 at D90). The excellent post-breakthrough infection neutralization activity to Delta, at similar Anti-S levels, is consistent with the Delta variant having caused the breakthrough infection. Anti-NC antibody response at *D*45 and D90 when comparing the rise in quantitative titres for breakthrough cases for the same subjects is shown in Fig. 2c.

The primary limitations of our data were that we did not have the necessary information to determine whether there were transmission chains with breakthrough infections, nor have we confirmed that these were Delta infections. However, our recently published work on the epidemiologic and genomic characterization of the same outbreak in community^8^ and HCWs^7^ shows that vaccination breakthroughs were nearly all due to the Delta variant during this outbreak. Our MnT assay data also confirms that neutralization titers increased specifically to delta, indicating that the infections were likely to have been Delta.

The other limitation is that we have combined an HCW cohort and a general cohort for estimating vaccine protection. While this could skew the estimates under normal conditions with HCW being at higher risk compared to the general population, the huge surge in community transmission during our study period minimizes any such bias. As published recently by our group, almost half the population was infected during the surge, with uniformly high exposures across social strata^8^.

Here, to overcome the limitation of lack of an unvaccinated HCW group in our main cohort, we have used a previously published cohort to approximate the likely infection rate in unvaccinated HCWs. The unvaccinated cohort had similarly high levels of exposure, as judged by baseline seropositivity levels. We have further shown that the very high seropositivity rates that were reached by the end of the Delta wave, were independent of most variables, including occupation^8^. This is consistent with other publications showing similar levels of seropositivity between HCWs and other groups, at different time points during the pandemic, possibly due to most infections being out of workplace^22–28^.

While we have identified transmission clusters in other published data^7^, the two situations are not directly comparable, with one being a serological diagnosis and the other being RT-PCR. Given the known relationships between RT-PCR positivity and humoral response, where seroconversion occurs in a subset of RT-PCR positive subjects, it seems reasonable to assume that infection sufficient to induce humoral response would also have been RT-PCR positive at some point^29^.

Within these limitations, our results have three important implications for the management of Delta outbreaks, some of which have already been confirmed by other data. First, neutralization of Delta variant by antibodies to non-Delta spike protein was greatly reduced such that levels greater than 1500 U/ml were required for high protection (Fig. 2a). This means that neither prior infection by non-Delta, nor most vaccines are individually sufficient for the path to herd immunity. Even with mRNA vaccines such as BNT162b2 mRNA, with much higher humoral responses, breakthrough infections become frequent during the declining phase of antibody concentration, and boosters that re-establish very high concentrations are required to break transmission^30,31^. This implies that mask-wearing should be an essential part of any rational COVID-19 control strategy, being agnostic to immune escape^32^. Second, given the reduced effectiveness of induced antibodies against non-delta spike protein, a single dose of vaccines should not be expected to confer useful protection against Delta variant infection. Thus, a second dose should be given early to the vulnerable population.

With the rapid transmission and immune evasion, delta affected every category. However, as there was much asymptomatic infection despite vaccination, HCWs should use non-vented masks with strict compliance and adherence to the safety guidelines to prevent transmission to patients. Hospitals have deployed major control measures including but not limited to strict mask-wearing (N95 and non-vented), minimal patient entry, strict social distancing norms, negative pressure, etc, and need to adhere to these strictly.

Last, our data indicate an urgency to explore routes towards more effective global use of vaccines. Even one dose of ChAdOx1-nCoV19 to previously infected subjects induced humoral immunity comparable to or better than two doses in naïve subjects. Thus, in populations with high seropositivity, the second dose to the low-risk population can be delayed and priority can be given to vaccinating more people. There may also be a benefit to using heterologous prime/boost strategies as shown by the ComCov study^19,33^, where using ChAdOx1-nCoV19 as prime followed by BNT162b2 mRNA boost provided statistically higher levels of humoral immunity comparable to homologous ChAdOx1 (12906 ELU/ml vs 1392 ELU/ml). Cellular immunity may be better by this approach and the decline may be slower, although this remains to be verified.

To conclude, our data show that vaccination breakthroughs in double vaccinated HCW were far more common during the Delta outbreak than previously reported^34^. Natural infection was seen to provide a strongly boosted response to vaccination such that it protected very effectively against the Delta variant.

## Methods

The study was approved by the ethics committees of Max Group of Hospitals, New Delhi, and CSIR-IGIB, New Delhi vide approval BHR/RS/PSH/MHIL/MHEC/IM 21-05, BHR/RS/MHG/MHIL/GGN/MHEC/IM 21-04, BHR/RS/MSSH/ MHIL/SKT-1/MHEC /NM 21-03, BHR/RS/PSH/MHIL/MHEC/IM 21-06, BHR/RS/PSH/MHIL/MHEC/IM 21-07 and CSIR-IGIB/IHEC/2019–20. Healthcare workers (HCWs) at the Max Hospital Group, who were to receive the ChAdOx1-nCoV19 vaccine, voluntarily enrolled after informed consent. The study was also registered; vide CTRI/2021/01/030782 at the Clinical Trial Registry of India as an observational cohort study.

Quantitative antibody response directed against the spike protein was measured at days 0, 7, 14, 28, 45, and 90, with qualitative antibody response on days 0,45, and 90 and neutralizing antibody response on days 28, 45, and 90. On day 45, we assessed subjects who had received their second dose at 28 ± 7 days and provided their sample at day 45 ± 3 days. Similarly, for data at 90 days, we included those who got their second dose up to 42 days and gave their sample at 90 ± 20 days. Herein, we analyzed data of 597 ChAdOx1-nCoV19 vaccine recipients. The timing between the first and second dose varied, but 485 received the second dose within 42 days of the first dose and most subjects received the second dose at 28 ± 3 days. Of these; 309 (52%) had already developed antibodies to SARS-CoV-2. HCWs were thus divided into two groups - seropositive (SP) and seronegative (SN), based on their serology (Anti-NC) status at baseline.

We were also following a second CSIR cohort of which the first phase had been completed^10^ with Phase 2 data collected during Jan-Feb 2021, the third phase collection was ongoing between May to June 2021^8^. Two separate cohorts were made for assessment of vaccination response and breakthrough for the HCWs as frontline workers had to necessarily receive the two doses at 28-day dosing interval in the initial period of vaccination program while the normal population from March-April onwards had delayed dosing interval and hence the cohort was amenable to monitoring for vaccination response against breakthrough infection when only a single dose was taken and also comprising of individuals who had not taken any vaccine to the date of sample collection and thus could be assessed for natural infection assessed both from history and serological response through nucleocapsid antigen-based qualitative antibody response. Data for information pertaining to participants was obtained through Google Forms from the participants.

### Sample collection

Blood samples (6 ml) were collected in K2 EDTA vials from each participant and antibodies to SARS-CoV-2 directed against the spike protein (S-antigen) were assayed using Elecsys Anti-SARS-CoV-2 S quantitative antibody detection kit (Roche Diagnostics), according to the manufacturer’s protocol. The kit has a clinical sensitivity of 98.8% and specificity of 99.98% with analytical specificity of 100%. Antibody levels >0.8 U/ml were considered seropositive. The range of the kit is from 0.4 U/ml to 250 U/ml. For samples with values of >250 U/ml appropriate dilutions were made. Samples were also assessed for qualitative antibody (Anti-NC) using the same manufacturer’s kit. The kit has a clinical sensitivity of 100% after 14 days and a specificity of 99.81%. Samples on Day 28, 45, and 90 were further tested for neutralizing antibody (NAB) response directed against the spike protein using GENScript cPass™ SARS-CoV-2 Neutralization Antibody Detection Kit (Genscript, USA), according to the manufacturer’s protocol^10^.

In this report, we have performed a microneutralization assay using a live virus. For this purpose, the SARS-CoV-2 virus belonging to the original Wuhan type strain and Delta strain were grown in VeroE6 cells and the infectious titer determined by Reed and Muench method. Aliquots of the virus were stored in a −80 °C freezer inside the BSL3 laboratory in single-use aliquots. Serum samples were heat-inactivated at 56 °C for 1 h and subsequently stored at a -80 °C freezer till assay. 10,000 VeroE6 cells from an exponentially growing culture were seeded per well in a 96-well plate for at least 20 h before the assay. On the day of assay, 2-fold serial dilutions of the serum were prepared in serum-free media (SFM) and the plate shifted to BSL3. Each serum dilution was mixed with 100 TCID50 of SARS-CoV-2 virus and incubated at 37 °C, 5% CO_2_ for 1 h. The mix of virus and serum dilutions were then allowed to infect the VeroE6 cell monolayer in the 96-well plate, for 1 h at 37 ^o^C, 5% CO_2_. The inoculum was discarded and growth media supplemented with 2% fetal calf serum overlaid on the monolayers. The plates were incubated at 37^o^C, 5% CO_2_ for 72 h. The cytopathic effect from the virus infection was scored visually using a phase-contrast microscope. The controls used included a positive control (no CPE) and a negative control serum (visible CPE) in addition to a mock-infection control (no CPE). The highest dilution of an experimental serum that inhibited the appearance of visible CPE was scored as the neutralization titre corresponding to that serum for the strain of virus used.

### Baseline seropositivity

To have baseline separation of seropositive and seronegative subjects, we utilized the CoI > =1, obtained from Roche Elecsys Kit (Anti-NC) as primary criterion or Anti-SARS CoV 2S Quantitative antibody levels >0.8 U/ml from Roche (Anti-S) as secondary criteria in parallel to classify a subject as baseline seropositive.

Thus, we didn’t rely upon one assay as the sole criterion. Combined Sensitivity of these two assays when calculated utilizing manufacturer-based indices, resulted in a sensitivity of 100% and specificity of 99.96 % using the following formula^35^:

1 $$	{\mbox{Sensitivity Combined in OR Parallel Testing}}\\ 	={{{{{\rm{Sens}}}}}}\,{{{{{\rm{of}}}}}}\,{{{{{\rm{A}}}}}}+{{{{{\rm{Sens}}}}}}\,{{{{{\rm{of}}}}}}\,{{{{{\rm{B}}}}}}-({{{{{\rm{Sens}}}}}}\,{{{{{\rm{of}}}}}}\,{{{{{\rm{A}}}}}}\,\times\,{{{{{\rm{Sens}}}}}}\,{{{{{\rm{of}}}}}}\,{{{{{\rm{B}}}}}})$$

2 $$	{\mbox{Specificity Combined in OR Parallel Testing}}\\ 	={{{{{\rm{Spec}}}}}}\,{{{{{\rm{of}}}}}}\,{{{{{\rm{A}}}}}}\,\times\,{{{{{\rm{Spec}}}}}}\,{{{{{\rm{of}}}}}}\,{{{{{\rm{B}}}}}}$$

Lower values available for sensitivity and specificity of this assay (Anti-NC) from literature is 95.5 and 96.2% respectively^36^. While for the quantitative assay (Anti-S) we could see a lower sensitivity of 97.2 and specificity of 99.95 %^37^. Utilizing these two indices we also observed a combined sensitivity and specificity of 99.88 and 96.15 % respectively. We note however that the likelihood of false negatives in this study is very low. Here we have tracked seroconversion in baseline seronegative subjects during a COVID-19 outbreak and the PPV of a positive test is near 100% in this scenario, because of high prior odds. In recently published work, we have also demonstrated the use of serology to estimate reinfection using three time points^8^.

### Statistical analysis

Data analysis and model development were carried out with visualization in MS-Excel 2016, OriginPro V2021, Stata 15 and R version 4.0.3. A non-Parametric Mann–Whitney test was utilized for SN and SP group comparison, for time-specific significant differences.

Relative Risk (RR) was calculated from the values observed for the respective group i.e. partially, fully, and unvaccinated participants^38^. Odds Ratio observed from the logistic model was converted to RR with background seropositivity of 87.3% in the unvaccinated control group^39–41^. Vaccine Effectiveness was then calculated as 1-RR.

### Reporting summary

Further information on research design is available in the Nature Research Reporting Summary linked to this article.

## Supplementary information

Supplementary Information

Reporting Summary

## Data Availability

All request for de-identified data should be send to corresponding author Dr Shantanu Sengupta at shantanus@igib.res.in as data pertains to HCWs with a detailed objective and proposal for usage. The data request can be sent after 90 days from the date of publication of this work and a signed agreement will be made with Max Hospital and IGIB for use of data.
