# Supplementary material for: High failure rate of ChAdOx1-nCoV19 immunization against asymptomatic infection in healthcare workers during a Delta variant surge
Source: Nat Commun. 2022 Apr 1;13:1726. doi: 10.1038/s41467-022-29404-3 (PMC8975928; doi:10.1038/s41467-022-29404-3)
Supplement: Supplementary file 1 — Supplementary Information [file 41467_2022_29404_MOESM1_ESM.pdf]

Supplementary information for

High failure rate of ChAdOx1-nCoV19 immunization against asymptomatic infection in healthcare workers during a Delta variant surge

Supplementary Note 1

Immunogenic Response with two doses of vaccine

Antibody response at 7 days in the Seropositive (SP) group was significantly higher than in the Seronegative (SN) group ($p<0.0001$) In fact, greater than eighty percent of subjects in the SN group did not develop any measurable response at day 7. This was corroborated at day 14 when subjects in the SN group started developing antibodies, though the difference was still significant amongst the two groups ($p<0.0001$). On day 28, before the second dose of vaccination, most of the individuals in the SN group had measurable antibody response (Figure 1A). At day 45, we assessed subjects who had received their second dose at 28±7 days and provided their sample at day 45±3 days. Similarly, for data at 90 days we included who got their second dose up to 42 days and gave their sample at 90±20 days. Interestingly, in the SP group, there was no further increase in the antibody levels after administration of the second dose and were observed to fall at day 90. However, in the SN group, the antibody levels kept rising after the second dose at day 45 but did not reach the levels of baseline seropositive group albeit at D_{90} the levels were stable in comparison to day 45 and were not observed to be falling unlike the SP group where nearly a two-fold decline was observed (Figure 1A).

Time for seropositivity for antibody naïve subjects from 1st dose of vaccine was 14 days for maximum subjects (~73%) in respect of quantitative antibody response, At day 7, there were 26 subjects (16%), who had a positive quantitative response on day 7. On day 28, 97% of subjects had a seroconversion, 6 subjects did not show seroconversion on day 28 after the first dose of vaccination.

Median levels for nAb (sVNT), which had already peaked at D_{28} remained stable at 97% in the SP group after the second dose when observed at day 45 and day 90. On the contrary, in the SN group the median level of the neutralizing antibody (sVNT) was 44% after the first dose which increased to 81% after the second dose. (Supplementary Fig. 1).

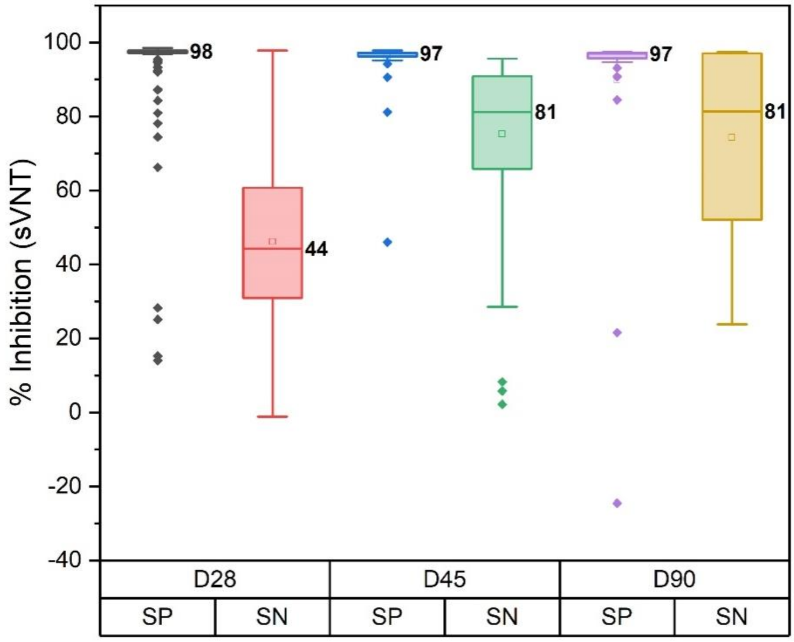

Supplementary Fig. 1: Neutralizing Antibody Response amongst baseline seropositive and seronegative participants. nAb response (sVNT) assay at D_{28}, D_{45} and D_{90} in SP and SN subjects (Values are rounded off). (Number of Samples For SP at D_{28}=128 , D_{45} =81, D_{90} = 32 and For SN at D_{28} =175 , D_{45} = 106, D_{90} = 110). Box plot is 25-75 range box, with median line and a square in the centre is mean. Whiskers are set at outliers with a coefficient of 1.5 and solid rhomboid shape are outliers.

Supplementary Note 2

Relaxed Criteria to assess breakthrough infection

To assess breakthrough, the criteria was;

Setting the dosing interval up to D_{42}, the subject should be Anti-NC negative at D_{45} and when followed to D_{90} should show a positive CoI i.e. CoI>=1. (D90 follow up included samples collected D_{70} onwards from 1st dose

However, under relaxed criteria, at D_{90} the CoI at D_{90} could be between 0.2 and 1, but, should show Anti-NC increase greater than two-fold and Anti-S increase greater than five-fold to qualify as a breakthrough (Supplementary Fig. 2). Combined sensitivity and specificity of the test with this criterion was 98% and 95.2% respectively.

With relaxed criteria breakthrough infection rate after two doses was 33.7%. A crude Relative Risk (RR) of 0.38 (95% CI 0.29-0.51%) corresponding to a protection efficacy of 62% (95% CI 49-71%) was observed.

An adjusted OR of 0.078 led to similar RR of 0.40 and PE of 60% (95% CI 42-76%) with age and gender to be insignificant confounders.

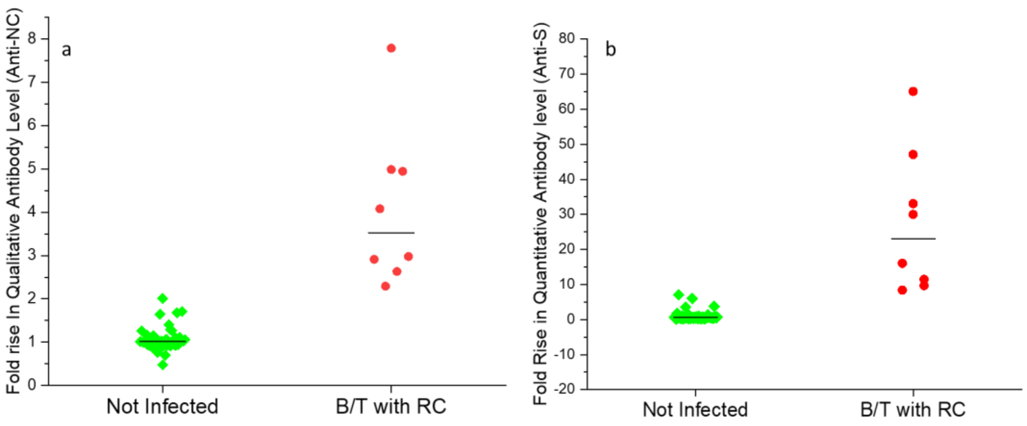

Supplementary Fig. 2: Qualitative and quantitative antibody response in breakthrough infection. Fold rise in qualitative (a) and quantitative antibody levels (b) for two groups; probable breakthrough with relaxed criterion (B/T with RC- red colour) and uninfected subjects (green colour). These subjects did not meet stringent criteria but were additionally considered as breakthrough for calculating protection efficacy, which fell from 70% to 60% upon their inclusion. Data represented is median lines with data points

Supplementary Method 1

Regression Model (Model based approach to evaluate RR and Protection Efficacy)

We performed a model-based approach (logistic regression) where we obtained an adjusted OR of 0.0518 with fully vaccinated subjects (two dose) and vaccination status being the only significant covariate with age and gender being insignificant ($p>0.05$). This OR corresponds to an adjusted RR of 0.30 resulting in vaccine effectiveness of 70% while we obtained unadjusted value of 71%. Similarly, for partially vaccinated (single dose), we obtained an adjusted OR of 0.136 which corresponds to an adjusted RR of 0.55 which is VE of 45% while our crude estimate was 41%. With partially vaccinated individuals also, age and gender were insignificant.

Crude RR for two and one vaccine doses in our data were 0.29 and 0.59 respectively, corresponding to about 71% and 41% vaccine effectiveness.

Supplementary Table 1 summarises the data for calculating the RR and VE

Cohort	Vaccine Status	Outcome-Had Infection	Outcome-No Infection	Total	RR (95%CI)	Protection Effectiveness (95% CI)	Adjusted RR based on Model# (95%CI)	Adjusted Protection Effectiveness # (95% CI)	Age (Median)	Gender M:F
CSIR	Non Vaccinated	117	17	134					21-61 (34)	100:34
CSIR	1 Dose	19	18	37	0.59 (0.43-0.81)	41% (19%-57%)	0.55 (0.27-0.84)	45% (16%-73%)	26-62 (52)	20:17
HCW	2 Dose	24	71	95	0.29 (0.20-0.41)	71% (59%-80%)	0.30 (0.17-0.48)	70% (52%-83%)	22-74 (39)	63:31*
HCW	2 Dose (relaxed criteria)	32	63	95	0.38 (0.29-0.51)	62% (49%-71%)	0.40 (0.24-0.58)	60% (42%-76%)	22-74 (39)	63:31*
HCW	NI + 2 dose	3	116	119	0.03 (0.009-0.088)	99.7% (99.12-99.991)				

Supplementary Table 1: Data for number of participants and outcomes to evaluate vaccine effectiveness

* Data of 1 participant not available for age and gender

Adjusted RR with CI was derived from OR and CI of model-based approach with background unvaccinated prevalence of 87.3% using this website https://clincalc.com/Stats/ConvertOR.aspx
