# Supplementary material for: High failure rate of ChAdOx1-nCoV19 immunization against asymptomatic infection in healthcare workers during a Delta variant surge
Source: Nat Commun. 2022 Apr 1;13:1726. doi: 10.1038/s41467-022-29404-3 (PMC8975928; doi:10.1038/s41467-022-29404-3)
Supplement: Supplementary file 2 — Reporting Summary [file 41467_2022_29404_MOESM2_ESM.pdf]

nature portfolio

Corresponding author(s): Debasis Dash, Sujeet Jha, Shantanu Sengupta

Last updated by author(s): Feb 18, 2022

Reporting Summary

Nature Portfolio wishes to improve the reproducibility of the work that we publish. This form provides structure for consistency and transparency in reporting. For further information on Nature Portfolio policies, see our Editorial Policies and the Editorial Policy Checklist.

Statistics

For all statistical analyses, confirm that the following items are present in the figure legend, table legend, main text, or Methods section.

n/a	Confirmed	
☐	☒	The exact sample size (*n*) for each experimental group/condition, given as a discrete number and unit of measurement
☒	☐	A statement on whether measurements were taken from distinct samples or whether the same sample was measured repeatedly
☐	☒	The statistical test(s) used AND whether they are one- or two-sided<br>*Only common tests should be described solely by name; describe more complex techniques in the Methods section.*
☐	☒	A description of all covariates tested
☒	☐	A description of any assumptions or corrections, such as tests of normality and adjustment for multiple comparisons
☐	☒	A full description of the statistical parameters including central tendency (e.g. means) or other basic estimates (e.g. regression coefficient) AND variation (e.g. standard deviation) or associated estimates of uncertainty (e.g. confidence intervals)
☒	☐	For null hypothesis testing, the test statistic (e.g. *F*, *t*, *r*) with confidence intervals, effect sizes, degrees of freedom and *P* value noted<br>*Give P values as exact values whenever suitable.*
☒	☐	For Bayesian analysis, information on the choice of priors and Markov chain Monte Carlo settings
☒	☐	For hierarchical and complex designs, identification of the appropriate level for tests and full reporting of outcomes
☒	☐	Estimates of effect sizes (e.g. Cohen's *d*, Pearson's *r*), indicating how they were calculated

Our web collection on statistics for biologists contains articles on many of the points above.

Software and code

Policy information about availability of computer code

Data collection	Google Forms
Data analysis	Data analysis was carried out with visualization in MS-Excel 2016, OriginPro V2021, Stata 15 and R version 4.0.3 and higher.

For manuscripts utilizing custom algorithms or software that are central to the research but not yet described in published literature, software must be made available to editors and reviewers. We strongly encourage code deposition in a community repository (e.g. GitHub). See the Nature Portfolio guidelines for submitting code & software for further information.

Data

Policy information about availability of data

All manuscripts must include a data availability statement. This statement should provide the following information, where applicable:

- Accession codes, unique identifiers, or web links for publicly available datasets
- A description of any restrictions on data availability
- For clinical datasets or third party data, please ensure that the statement adheres to our policy

All request for de-identified data should be send to corresponding author at shantanus@igib.res.in as data pertains to Health Care Workers with a detailed objective and proposal for usage. The data request can be send after 90 days from the date of publication of this work and a signed agreement will be made with Max Hospital and IGIB for use of data.

Field-specific reporting

Please select the one below that is the best fit for your research. If you are not sure, read the appropriate sections before making your selection.

☒ Life sciences ☐ Behavioural & social sciences ☐ Ecological, evolutionary & environmental sciences

For a reference copy of the document with all sections, see nature.com/documents/nr-reporting-summary-flat.pdf

Life sciences study design

All studies must disclose on these points even when the disclosure is negative.

Sample size	We enrolled based on a voluntary enrollment for an observational study of 597 HCW's, who gave their samples for serological monitoring from different sites for MAX group of Hospitals in Delhi-NCR. Sample size was not calculated apriori as study was designed to have maximum enrollment possible.
Data exclusions	At D7 in Baseline SN subjects five subjects had data discordance; with 4 having having very high quantitative Ab levels but no data was available beyond d7 collection, so entire entry was removed. One subject had high quant at D7 but went to negative levels immediately at D14 and no data was available for thereafter collection. Hence, these 5 entries were removed from baseline SN subjects
Replication	This was a longitudinal cohort of HCW's that was being followed and technical sample replication was done for 10 percent of the samples.
Randomization	Not Applicable as Observational Study
Blinding	Not Applicable as it is an observational study and no blinding was done

Reporting for specific materials, systems and methods

We require information from authors about some types of materials, experimental systems and methods used in many studies. Here, indicate whether each material, system or method listed is relevant to your study. If you are not sure if a list item applies to your research, read the appropriate section before selecting a response.

Materials & experimental systems

n/a	Involved in the study	
☐	☒	Antibodies
☐	☒	Eukaryotic cell lines
☒	☐	Palaeontology and archaeology
☒	☐	Animals and other organisms
☐	☒	Human research participants
☐	☒	Clinical data
☒	☐	Dual use research of concern

Methods

n/a	Involved in the study	
☒	☐	ChIP-seq
☒	☐	Flow cytometry
☒	☐	MRI-based neuroimaging

Antibodies

Antibodies used	Elecsys Anti-SARS-CoV-2 S quantitative antibody detection kit (Roche Diagnostics),<br><br>Qualitative antibody (Anti-NC) SARS-CoV-2 NC antigen- Elecsys Anti-SARS-CoV-2 kit (Roche Diagnostics)<br><br>Neutralizing antibody (NAB) (sVNT) response directed against the spike protein using GENScript cPass™ SARS-CoV-2 Neutralization Antibody Detection Kit (Genscript, USA)
Validation	As per manufacturer's data sheet , controls were run as required as per manufacturer protocol

Eukaryotic cell lines

Policy information about cell lines

Cell line source(s)	Vero E6 cell line-NCCS Pune
Authentication	We were unable to undertake authentication due to the lockdown
Mycoplasma contamination	The cells were regularly checked for mycoplasma contamination by PCR amplification from culture supernatant

Commonly misidentified lines (See ICLAC register)	Commonly misidentified line is Vero cell line

Human research participants

Policy information about studies involving human research participants

Population characteristics	597 HCW's, who gave their samples for serological monitoring from different sites for MAX group of Hospitals in Delhi-NCR. CSIR-Phenome India Cohort Delhi Participants of Phase 3 (Details of CSIR -Cohort are available at eLife 2021;10:e66537 DOI: 10.7554/eLife.66537 and https://www.medrxiv.org/content/10.1101/2021.06.02.21258076v3)<br><br>Briefly, the HCW cohort contained 597 ChAdOx1-nCoV19 vaccine recipients. The timing between the first and second dose varied, but 485 received the second dose within 42 days of the first dose and most subjects received the second dose at 28±3 days. Fifty-two percent of subjects (n=309) had been previously infected with SARS-CoV-2, based on the presence of antibodies to SARS-CoV2 proteins (spike, anti-S or nucleocapsid, anti-NC) at D0, the day of the first dose. There was a robust immunogenic response to two doses of vaccine, irrespective of prior infection (Figure 1C). Time for seropositivity for antibody naïve subjects from 1st dose of vaccine was 14 days for maximum subjects (~73%) in respect of quantitative antibody response. At day 7, there were 26 subjects (16%), who had a positive quantitative response. On day 28, 97% of subjects had a seroconversion, while 6 subjects did not have seroconversion even on day 28 after the first dose of vaccination.<br>To have a surrogate matching cohort for calculating infection rates and vaccine effectiveness, the next best matching cohort available to us was the CSIR -Cohort, for which had earlier done two rounds of serosurvey and reported previously8. This cohort is not exactly a general population cohort but comprised of researchers, students, scientists, and their family members, many of whom were working as frontline workers in covid pandemic mitigation and testing work. The seropositivity in Delhi during that period for unvaccinated subjects in our cohort was 87.3 percent and that itself we took as a non-vaccinated cohort. .<br><br>Briefly, Phase 3 of CSIR cohort for Delhi was majorly conducted in May-June 2021 matching the period of HCW cohort collection at D90. There were 729 participants during this period of which 637 were seropositive (87.4%). The Phase 2 was conducted in January and February of 2021. Of 729 participants in Phase 3, 134 participants had also provided samples in previous phase 2 and were seronegative during that collection time point. Of these 117 were found to be positive in phase 3 (87.3%) and thus were infected during this period.<br><br>Of these 134; 100 were male and 34 female with age varying between 21 to 61 years and a median of 34 years.<br><br>For fully vaccinated HCW's of 95 participants; 63 were male and 31 were females. Age varied between 22 to 74 years with a median age of 39 years
Recruitment	We recruited based on a voluntary enrollment as an observational cohort. No self selection bias was present.
Ethics oversight	MAX Hospital Ethics Committee and IGIB-IHEC

Note that full information on the approval of the study protocol must also be provided in the manuscript.

Clinical data

Policy information about clinical studies

All manuscripts should comply with the ICMJE guidelines for publication of clinical research and a completed CONSORT checklist must be included with all submissions.

Clinical trial registration	CTRI/2021/01/030782
Study protocol	Available on CTRI site at http://ctri.nic.in/Clinicaltrials/advancesearchmain.php
Data collection	At max group of hospital sites, we would do antibody testing at day 0 (vaccination day or pre-vaccination) followed by 7, 14, 28, 45, 90th day of vaccination sample collection for HCW and was based on a voluntary enrollment.<br>This study would involve a brief questionnaire asking about the health status of the participants related to COVID-19 and side-effects post-vaccination.<br>The time period of collection is available in Figure 1A
Outcomes	It was an observation study for enrollment of HCW and monitoring their antibody response post vaccination at specified days. Hence, no specific outcome was designated
